# Future Prospects in the Diagnosis and Management of Localized Prostate Cancer

**DOI:** 10.1155/2013/347263

**Published:** 2013-09-14

**Authors:** Ahmet Tefekli, Murat Tunc

**Affiliations:** ^1^Department of Urology, Bahcesehir University School of Medicine, 34353 Istanbul, Turkey; ^2^Department of Urology, Istanbul Faculty of Medicine, Istanbul University, 34390 Istanbul, Turkey

## Abstract

Prostate cancer (PCa) is the commonest visceral cancer in men worldwide. Introduction of serum PSA as a highly specific biomarker for prostatic diseases has led to a dramatic increase in the diagnosis of early stage PCa in last decades. Guidelines underline that benefits as well as risks and squeals of early diagnosis and treatment should be discussed with patients. There are several new biomarkers (Pro-PSA, PCA-3 test, and TMPRSS2-ERG) available on the market but new ones are awaited in order to improve specificity and sensitivity. Investigators have also focused on identifying and isolating the gene, or genes, responsible for PCa. Current definitive treatment options for clinically localized PCa with functional and oncological success rates up to 95% include surgery (radical prostatectomy), external-beam radiation therapy, and interstitial radiation therapy (brachytherapy). Potential complications of overdiagnosis and overtreatment have resulted in arguments about screening and introduced a new management approach called “active surveillance.” Improvements in diagnostic techniques, especially multiparametric magnetic resonance imaging, significantly ameliorated the accuracy of tumor localization and local staging. These advances will further support focal therapies as emerging treatment alternatives for localized PCa. As a conclusion, revolutionary changes in the diagnosis and management of PCa are awaited in the near future.

## 1. Introduction

Prostate cancer (Pca) is the most common noncutaneous malignancy and the second leading cause of cancer death in men [1]. According to very recently published statics, cancers of the prostate, lung and bronchus, and colorectum will account for about half of all newly diagnosed cancers among men while prostate cancer alone is underlined to account for 29% (241,740) of incident cases [1]. Furthermore, cancers of the lung and bronchus, prostate, and colorectum in men will continue to be the most common causes of cancer death [1].

In the United States, 90% of men with Pca are older than 60 years, diagnosed by early detection with the serum prostate-specific antigen (PSA) blood test, and have disease believed to be confined to the prostate gland [2]. Considering these factors as well as the sociocultural position of this group of men, the treatment of the localized Pca stands out as a major health problem. 

Current treatment options for clinically localized Pca include active surveillance (AS), surgery (radical prostatectomy), external-beam radiation therapy, and interstitial radiation therapy (brachytherapy) [3]. Highly satisfactory success rates up to 95% are being reported using a single or a combination of these treatment modalities [3]. 

## 2. Screening and Early Detection

Improved treatment techniques as well as earlier diagnosis in recent years have certainly led to better results [3]. However, early diagnosis and/or early treatment of Pca has interestingly not improved the Pca specific survival or overall survival from Pca [4]. On the other hand, there is an everyday increasing number of publications dealing with new markers to detect Pca in the early stage [5]. Although PSA is a prostate specific marker, it is generally agreed that the PSA test is not a perfect test for finding Pca in its early phase. In order to improve the sensitivity and specificity of serum PSA, several PSA derivates and isoforms are being used [5]. 

Prostate Health Index (Phi index: *Phi index: [−2] proPSA/fPSA × PSA* ^1/2^) has recently been suggested as a useful tool by Catalona et al., especially in men with a serum PSA 2–10 ng/mL [6]. Previous studies have shown that elevated (pro-PSA/free PSA) ratios are associated with aggressive pathological features and decreased biochemical disease-free survival after radical prostatectomy [7]. A new automated tool using the [−2]proPSA assay with a percent-free PSA-based artificial neural network was reported to be capable of detecting Pca and more aggressive diseases with higher accuracy than total PSA or percent-free PSA alone [8]. In a recent prospective cohort of men enrolled into active surveillance for PCa, serum and tissue levels of pro-PSA at diagnosis were associated with the need for subsequent treatment [9]. The authors suggested that the increase in the ratio of serum pro-PSA to percent-free PSA might be driven by increased pro-PSA production from “premalignant” cells.

Despite the worldwide popularity of PSA, there are still debates going around it [10]. First of all, it is questioned whether PSA-based screening decreases prostate cancer-specific or all-cause mortality [3, 11]. In the recently published PIVOT study which was performed among men with localized Pca detected during the early era of PSA testing, radical prostatectomy did not significantly reduce all-cause or Pca mortality, as compared with observation, through at least 12 years of followup [11]. Furthermore, in another recently published prospectively randomized study called the Prostate, Lung, Colorectal, and Ovarian (PLCO) Cancer screening Trial, it was also concluded that Pca mortality was not significantly different between the PSA (and DRE) screened and control groups [12]. On the other hand, data from the “European Randomized Study of Screening for Prostate Cancer” (ERSPC) suggests that PSA-based screening reduced the rate of death from prostate cancer by 20% [13]. Based on the results of these two large randomized trials, most of the major urological societies conclude that at present widespread mass screening for Pca is not appropriate [14]. According to the European Association of Urology Guidelines, early detection (opportunistic screening) should be offered to the well-informed men [14]. 

The American Cancer Society (ACS) recommends that men who are over 50 years of age and who are expected to live at least 10 more years should have a chance to make an informed decision with their health care provider about whether to be screened for Pca or not [15]. The decision should be made after getting information about the uncertainties, risks, and potential benefits of Pca screening. The ACS interestingly underlines that men should not be screened unless they have received this information [15]. According to the ACS, this discussion should take place starting at age 40 for men at high risk of developing Pca. This includes African Americans and men who have a first-degree relative (father, uncle, brother) diagnosed with Pca at an early age (younger than age 65) [15]. And finally, after this discussion, those men who want to be screened are suggested to be tested with the serum PSA as well as digital rectal exam (DRE) [15]. The ACS also suggests that men without symptoms of Pca who do not have a 10-year life expectancy should not be offered testing since they are not likely to benefit because it is generally considered that prostate cancer growth is slow [15]. However, a recently published report from three decades of followup of the natural history of Pca underlines that, although localized Pca most often has an indolent course, local progression and distant metastasis can develop over the long term, even among patients considered to be at low risk at diagnosis [16]. In this study, 38 (17%) of the 223 untreated men with localized Pca died because of prostate cancer after 32 years of followup [16]. The authors observed 90 (41.4%) local progression events and 41 (18.4%) cases of progression to distant metastasis, and these findings further complicate discussions around screening [16]. 

There are even slight disparities among guidelines declared by the same country, the USA. In the very recent annual meeting of the American Urological Association, guidelines on the early detection of Pca have been presented and some small changes are underlined [17]. According to this very recent declaration, in men aged 40–54 at average risk for the disease, the guidelines recommends that screening, as a routine practice, should not be encouraged. The Guidelines Committee underlines that evidence for the benefit for screening in this age range was limited while the quality and strength of the evidence regarding the harms of screening was high. In addition, routine screening were not recommended in men over the age of 70 or those with less than a 10-year life expectancy. However, the AUA guidelines acknowledged that some men over the age of 70 in excellent health might benefit from screening. In this setting, the guidelines suggest that a discussion of the unique risks and benefits of screening in older men occur. The same guidelines also point out that the highest quality evidence for benefit (defined as lower prostate cancer mortality) of screening was found in men aged 55 to 69 years. In men aged between 55 and 69 years, the guidelines strongly recommended shared decision making and screening based on a man's values and preferences. The only difference in the new guidelines is that they now recommend biennial screening to reduce the potential harms of screening. And interestingly the new AUA Guidelines stand out to be in disagreement with the US Preventive Services Task Force in recommendation against Pca screening in all men, regardless of age or risk, without even considering a discussion of the risks and benefits of screening. The U.S. Preventive Services Task Force recommends against PSA-based screening for Pca as a grade D recommendation and this recommendation applies to men in the general U.S. population, regardless of age [18]. However, the AUA continues to support a man's right to be tested for Pca and to have the insurance pay for it, if medically necessary [17]. 

Another debate going on around early detection is that we still do not clearly know the consequences of the treatment of early Pca detected by PSA screening. As mentioned above, it is evident that PSA-based screening results in reduction in prostate cancer-specific mortality, but it is associated with harm related to subsequent evaluation and treatments, some of which may be unnecessary [11, 18]. Therefore, informing a potential patient about the risks and benefits of screening is highly suggested and individual risk assessment is supported.

## 3. New Biomarkers

Research for a new marker has focused on serum-based, tissue-based and urine-based markers [19–21]. Despite extensive research efforts, very few biomarkers of Pca have been successfully implemented into clinical practice today and serum PSA test is still the most important biomarker for the detection and followup of Pca. Numerous studies of serum-, tissue, and urine-based prostate cancer biomarker candidates have been presented the last ten years [19–21]. It is generally accepted that unmet biomarker for prostate cancer should be addressed to distinguish BPH from Pca, to detect the aggressive forms from the indolent cases, and to identify the metastatic cancer predictors. However, biomarkers for identifying the most aggressive subsets of this malignancy are still missing. Briefly, PSA isoforms, pHi, and other combinations seem to be promising among serum-based biomarker. Tissue-based biomarkers are classified as diagnostic dyes, which are generally used to differentiate cancer with PIN and atypia, and prognostic biomarkers, which are usually determined on prostatic tissue using different techniques and are far from being a screening tool [20]. Out of urine-based marker, PCA-3 test is already in current daily practice and highly satisfactory results are being reported [21].

PCA-3 test has recently been approved by FDA [21, 22]. PCA-3 test is a urine-based marker, in which urine collected after a rectal exam and prostatic massage is highly specific for Pca, and is not affected by prostate volume and chronic prostatitis. It is also considered to be helpful in deciding rebiopsies and in the followup of patients under AS [22]. *PCA3 *is a non coding RNA and the marker most specific to Pca that is clinically available to date. *PCA3* RNA expression is restricted to the prostate, and it is not expressed in any other normal human tissue or tumor. PCA3 RNA is highly overexpressed in 95% of tumors compared to normal or benign hyperplastic prostate tissue. Hessels et al. reported a median of 66-fold upregulation of *PCA3* in PCa tissue compared with normal prostate tissue [23]. 

To assess the probability of PCa detection on prostate biopsy, the quantitative PCA3 score was developed. The score is defined as the ratio (*PCA3 *mRNA/PSA mRNA X1,000), meaning that *PCA3* expression is normalized with PSA expression serving as a prostate housekeeping gene [23]. Since a PCA3 score of 35 yielded the greatest diagnostic usefulness, demonstrating the optimal balance between specificity and sensitivity, it was adopted as a cut-off score. The average sensitivity and specificity of the PCA3 urine test is relatively high at 66% and 76%, respectively, versus 47% specificity for serum PSA [24]. 

To increase the predictive accuracy of the biopsy outcome and identify men at risk for PCa, novel biopsy nomograms were created, including that for *PCA3*. Auprich et al. have recently assessed the accuracy of the previously reported *PCA3-*based nomogram in a large European cohort of men [25]. The nomogram helped identify PCa in 255 of 621 men (41.1%) [25]. 

Another promising marker looks for an abnormal gene change called *TMPRSS2:ERG* in prostate cells [26, 27]. Gene alterations involving androgen regulated *TMPRSS2 *and *ETS *transcription factor genes were identified in prostate cancer patients. *TMPRSS2 *fusion with the *ETS *family member, an *ERG*, is the predominant variant in approximately 40% to 70% (about 50%) of patients with PCa. *ERG *is regarded as a key PCa oncogene. Considering the high prevalence of PCa, *TMPRSS2:ERG* fusion is the most common genetic aberration described to date in human solid tumors [27]. The cells to be tested are found in urine collected after a rectal exam. This gene change is found in more than 50% of all localized prostate cancers [26]. It is rarely found in the cells of men without prostate cancer. TMPRSS2-ERG has a specificity of 97% and sensitivity of 96%, and currently it is commercially available for clinical use in the US, and Europe [26]. 

In a very recent PubMed and Web of Science database search of the peer reviewed literature on urine-based testing for Pca, in an attempt toward the detection of Pca in urine, investigators have identified PCA3 and TMPRSS2:ERG fusion transcripts as promising RNA markers for cancer detection and possibly prognosis [28]. 

## 4. Genetics and Risk Assessment

In relation to investigations on genetic-based biomarkers, the key to curing Pca will ultimately come from an understanding of the genetic basis of this disease. Therefore, investigators have focused on identifying and isolating the gene, or genes, responsible for Pca [29]. Several high-penetrance genetic variants have been identified in many genetic linkage and genome-wide association studies around the world [29]. Many polymorphisms in genes, such as ELAC2 (locus HPC2), RNase L (locus hereditary prostate cancer 1 gene (HPC1s)), and MSR1, have been recognized as important genetic factors that confer an increased risk of developing Pca in many populations [29]. Tests to find abnormal Pca genes could also help identify men at high risk who would benefit from more intensive screening or from chemoprevention trials. Creation of a personalized panel of single-nucleotide polymorphisms (SNP) biomarkers may be important for the early and accurate detection of this cancer [30]. As a result, the need for a good biomarker is required to detect Pca earlier and to provide tools to follow patients during the early stages of the cancer. Furthermore, the use of a biomarker combination panel needs to be considered, in order to increase diagnostic accuracy. 

 A big enigma now facing men with prostate cancer and their doctors is figuring out which cancers are likely to stay within the gland and which are more likely to grow, spread, and definitely need treatment. In other words, world-wide accepted criteria to define low-intermediate-high risk prostate cancer are needed. The definition of high risk, which is still a matter of debate, was classically defined by Bastian et al. as any combination of the following factors: a prostate-specific antigen (PSA) score >20 ng/mL, a Gleason score of 8–10, or clinical stage T2C or greater [31]. Patients with high risk disease, which accounts for ≤15% of all new diagnoses, are more or less the focus of radical prostatectomy, either as mono- or multimodel therapy concept [31].

The potential complications of overdiagnosis and overtreatment have resulted in arguments about screening and introduced a new management approach called active surveillance as summarized above. The recent discovery of more than the 30 so-called prostate cancer susceptibility genes suggests the possibility of targeted screening of those men who have the highest risk of developing the aggressive form of Pca [28–30]. This could eventually help us to tell which men need treatment and which might be better served by active surveillance. For example, the product of a gene known as *EZH2* seems to appear more often in advanced prostate cancers than in those at an early stage [32]. Further studies will also be performed to try to block, or modify, the offending genes in order to prevent or alter the progression of prostate cancer. 

## 5. Promising New Medical Treatment Options

On the other hand “Gene Therapy,” which is a process of introducing genetically engineered material, usually DNA, into the body, is an evolving treatment option for Pca, but currently for advanced disease [33]. In a recently published report, experts reviewed the progress being made in gene therapy for Pca [33]. Overall, most of the more than 90 clinical protocols using gene therapy in Pca cancer patients in the National Institutes of Health database used adenoviral vectors [33]. While adenoviral gene therapy strategies in Pca patients were proved to be safe thus far, scientists are still struggling to identify which approaches should be considered and improved. However, experts must first conduct randomized, well-controlled Phase 3 trials, and that point has not yet been reached [33, 34]. 

Virus therapy, also known as “oncolytic virus therapy,” is a new potential treatment strategy for advanced prostate cancer patients and is still in the early stages of investigation. A virus chosen to treat cancer is called an oncolytic virus, and once it is introduced to the prostate cancer cells, it replicates and kills tumor cells selectively [35]. The progeny viruses produced within the cancer cells are then released, and they spread and infect other cancer cells that surround it. This cycle continues and results in the killing of more and more cancer cells. Because oncolytic viruses are not able to replicate in healthy cells, normal tissue is not damaged [35, 36]. Experts believe that the development of oncolytic virus therapy will eventually lead to a promising treatment option for men who have Pca, but ethical issues prevent these investigations among men with localized Pca.

There are also two “vaccines” commercially available for the management of advanced stage Pca [37, 38]. However, vaccines to prevent the disease in the early stage are awaited. Unlike vaccines against infections like measles or mumps, these currently available vaccines are designed to help treat, not prevent, prostate cancer. An example of this type of vaccine is Sipuleucel-T (Provenge), which has received FDA approval. Although clinical experience with this vaccine is limited, it has been shown to improve survival in patients whose cancer has become resistant to hormones. However, the cost of each treatment course is enormous—about $100,000, because doses of Sipuleucel-T are unique and individually prepared for each patient. 

The other available Pca vaccine (PROSTVAC-VF) uses a virus that has been genetically modified to contain PSA but is still investigational. The patient's immune system should respond to the virus and begin to recognize and destroy cancer cells containing PSA. Early results with this vaccine have been promising [38]. Several other prostate cancer vaccines are also in development.

There are great advances in the medical treatment of advanced and metastatic disease, but this topic is out of the scope of this review. However, once the efficacy of these new compounds for advanced and incurable disease has been established, these agents may be explored as an adjuvant and neoadjuvant treatment in order to increase the chance of cure for localized disease. And abiraterone, especially, a new compound used for metastatic disease may be offered to patients with localized prostate cancer who refuse radical treatment options. 

## 6. Life Style and Diet

Life style and dietary alterations are also believed to alter the progression of prostate cancer [39]. Observational evidence show that there is a relationship between the so-called energy balance factors (i.e., diet, physical activity, and body weight) and risk of cancer recurrence as well as mortality in cancers of the breast, prostate, colon, and, perhaps, other cancers. Furthermore, individuals who make favorable changes in these lifestyle factors after cancer diagnosis feel better, experience less fatigue, and may possibly even decrease risk of cancer recurrence [39]. Other lifestyle behaviors, such as smoking and alcohol consumption, have also been linked to the development of common cancers and may have important health consequences for cancer survivors. An interesting study has shown that in men with a rising PSA level after surgery or radiation therapy, drinking pomegranate juice seemed to slow the time it took the PSA level to double [40]. Larger studies are now trying to confirm these results. Supporting the role of pomegranate as a strong antioxidant, investigators highly suggest the use of pomegranate extracts in the therapy of erectile dysfunction, benign prostatic hyperplasia, and Pca [41]. Therefore, patients with localized Pca may also be advised to consume pomegranate juice. 

Some encouraging early results have also been reported with flaxseed supplements. Studies indicate that enterolactone and enterodiol, mammalian lignans derived from dietary sources such as flaxseed, sesame seeds, kale, broccoli, and apricots, may impede tumor proliferation by inhibiting activation of nuclear factor kappa B (NF*κ*B) and vascular endothelial growth factor (VEGF) [42]. One randomized controlled study in men with early Pca before surgery found that daily flaxseed seemed to slow the rate at which Pca cells multiplied [43]. More research is needed to confirm this finding. Another study found that men who chose not to have treatment for their localized Pca may be able to slow its growth with intensive lifestyle changes [44]. The men ate a vegan diet (no meat, fish, eggs, or dairy products) and exercised frequently, and the authors observed a slight diminishment in the serum PSA levels after one year. However, it is not known if this effect will last longer since the report only followed the men for 1 year. 

## 7. Advances in Diagnosis

Researchers also keep on searching how to improve the diagnostic accuracy of transrectal ultrasound guided biopsy (TRUS-bx), which currently the basic way to diagnose Pca [45]. It is well-known that standard ultrasound may not detect some areas containing cancer. Therefore, a newer approach is to measure blood flow within the gland using a technique called “*color Doppler ultrasound*” since tumors often have more blood vessels around them than normal tissue. It may make prostate biopsies more accurate by helping to ensure that the right part of the gland is sampled. An even newer technique may enhance color Doppler further, called “contrast enhanced color Doppler US.” It involves first injecting the patient with a contrast agent containing microbubbles. Promising results have been reported, but more studies are needed before its use becomes common [45]. 

Apart from a possible role in the diagnosis of PCa, elasticity imaging techniques may monitor high intensity focused ultrasound (HIFU) results in prostate cancer, because HIFU-ablated lesions are stiffer than the surrounding normal untreated tissue [45]. Promising results have recently been published, but further clinical trials are needed before this application can be considered established.

There are increasing number of publications regarding the use of MRI in the diagnosis of prostate cancer [46, 47]. Magnetic resonance (MR) imaging currently plays a pivotal role in pretreatment assessment of prostate cancer. Multiparametric MR imaging, a combination of anatomic and functional MR imaging techniques (diffusion-weighted imaging, dynamic contrast material-enhanced imaging, and MR spectroscopy), significantly improves the accuracy of tumor localization and local staging [48]. MRI anatomic imaging with spectroscopic evaluation analyzes cellular metabolites within the prostate and their changes in PCa [49]. In the prostate, choline and citrate are the important metabolites [49]. Choline is an important component of cell membranes, integrated into the phospholipid bilayer. Prostate malignancy is hypothesized to lead to increased choline because of increased cell proliferation. Citrate is a component of the citric acid cycle that normally accumulates within the glandular ducts formed by prostate epithelial cells. Prostate malignancy is thought to lead to decreased choline levels by means of increased tumor metabolic activity and decreased glandular differentiation [49]. An accuracy up to 90% has been reported with dynamic contrast-enhanced MRI in detection and localization of prostate [48]. Therefore, MRI can especially help to guide prostate biopsies in men who previously had negative TRUS-guided biopsies [47, 49, 50]. In a very recent paper, the role of “MRI-targeted TRUS-guided transperineal fusion biopsy” in the diagnosis of Pca was evaluated in 347 consecutive patients [50]. The majority of these patients had a history of negative TRUS-guided biopsies. In the study, all patients underwent multiparametric (mp) MRI at 3T and received systematic stereotactic prostate biopsies plus MRI-targeted TRUS-guided biopsies in case of MRI abnormalities [50]. The investigators were able identify Pca in 58% of the samples and concluded that MRI-targeted TRUS-guided transperineal fusion biopsy provides high detection rates of clinically significant tumors. However, they also underline that this technique still has some limitations, and therefore systematic biopsies should currently not be omitted [50]. Similarly, a median Pca detection rate of 42% has been reported in a recent meta-analysis [51].

Another advantage offered by new MRI technologies is that anatomic MR imaging provides highly accurate local staging information, particularly about extraprostatic extension and seminal vesicle invasion for pretreatment planning (especially for external beam radiotherapy and brachytherapy) [48]. The dominant intraprostatic tumor and local recurrence in the prostatectomy bed can be better localized with multiparametric MR imaging for dose painting [48]. MRI can also be used in early posttreatment evaluation after brachytherapy [48]. 

Furthermore, MRI is becoming more important in the followup of patients under AS [52]. *Enhanced MRI* may also help us to detect lymph nodes that contain cancer better than conventional CT and MRI. A newer type of positron-emission tomography PET scan that uses radioactive carbon acetate instead of FDG may also be helpful in detecting Pca in different parts of the body, as well as helping to determine if treatment has been effective [49]. Studies of this technique are now in progress [49]. 

## 8. Active Surveillance

In addition to advances in the screening, prevention, and diagnosis of Pca, researchers spent a big effort on treatment options and their comparative results. Despite a large number of publications on this area, little is known about the relative effectiveness and harms of treatments because of the paucity of randomized controlled trials. Recently, the Departments of Veterans Affairs/National Cancer Institute/Agency for Healthcare Research and Quality Cooperative Studies Program Study  no. 407:  Prostate Cancer Intervention Versus Observation Trial (PIVOT) reported a multicenter randomized controlled trial, initiated in 1994, comparing *radical prostatectomy* with *watchful waiting* in men with clinically localized Pca [11]. In this large study, a total of 13.022 men with prostate cancer at 52 US medical centers were considered for potential enrollment and a total of 731 men agreed to participate and were randomized [11]. PIVOT enrolled an ethnically diverse population representative of men diagnosed with Pca in the United States. During the median followup of 10.0 years, 171 of 364 men (47.0%) assigned to radical prostatectomy died, as compared with 183 of 367 (49.9%) assigned to observation. Among men assigned to radical prostatectomy, 21 (5.8%) died from prostate cancer or treatment, as compared with 31 men (8.4%) assigned to observation. The effect of treatment on all-cause and Pca mortality did not differ according to age, race, coexisting conditions, self-reported performance status, or histologic features of the tumor. Radical prostatectomy was associated with reduced all-cause mortality among men with a PSA value greater than 10 ng per milliliter and possibly among those with intermediate-risk or high-risk tumors. As a conclusion, the authors state that radical prostatectomy did not significantly reduce all-cause or Pca mortality, as compared with observation, among men with localized prostate cancer detected during the early era of PSA testing [11]. 

However, in a previous paper from the Scandinavian prostate cancer group, comparing radical prostatectomy and watchful waiting, it was concluded that radical prostatectomy reduces Pca mortality and risk of metastases with little or no further increase in benefit 10 or more years after surgery [53]. Comparison of the data sample of eligible men declining PIVOT participation as well as to men enrolled in the Scandinavian trial indicated that PIVOT enrollees are representative of men being diagnosed and treated in the United States and are quite different from men in the Scandinavian trial [11, 53]. 

 Basically taking the results of the PIVOT study and the Scandinavian study as well as the natural history of prostate cancer into consideration, a relatively new management concept called “active surveillance” has been introduced into the practice. In this new management concept, definitive treatment options of localized prostate cancer are deferred until certain level of progression with the patient under close control with serial serum PSA analyzes and repeats TRUS-guided prostate biopsies [3]. Recent findings suggest that detailed MRI studies as well as new prostate cancer markers such as PCA-3 test are helpful in the followup of patients under AS and especially in defining progression which is an absolute indication for the timing definitive treatment [47, 49]. 

AS means deferring treatment initially for a growing proportion of men diagnosed with low-risk (i.e., low volume, stage, and grade) Pca [54]. However, there is no worldwide accepted consensus on defining exact criteria in order to offer active surveillance to men with Pca [55]. Different institutions use different criteria to include men into active surveillance protocol [55]. In general, patients with PSA < 10, Gleason score <3 + 3 or 3 + 4, and less than 3 positive cores on TRUS biopsy are candidates for active surveillance. Men under active surveillance are followed carefully with serial PSA assessments, repeated biopsies, and in some cases other tests intended to identify early signs of progression (such as MRI and biomarkers). 

The term “active surveillance” has supplanted “watchful waiting,” but the two are not synonymous. The latter term is generally applied to older men with significant comorbidity, who were advised to defer treatment unless symptoms of advanced disease developed, at which point palliative androgen deprivation could be offered. Active surveillance, on the other hand, rests on the presumptions that the lead time from diagnosis to clinical progression is usually long for low risk disease and that at the first signs of higher-risk disease the cancer can be treated, within the window of opportunity for cure [54, 55]. 

## 9. Radical Prostatectomy

Although new developments are being waited to be introduced into practice about biomarkers and genetics, the surgical treatment (radical prostatectomy), which is currently considered to be the gold standard in the management of localized Pca, has almost achieved its excellence since its first anatomical description by Walsh more than 30 years ago [3, 56]. The overall 25-year progression-free, metastasis-free, and cancer-specific survival rates after anatomical radical prostatectomy were 68%, 84%, and 86%, respectively, although there were significant differences in treatment outcomes between men treated in the pre-PSA and PSA eras. In each era, there were significant differences in progression-free, metastasis-free, and cancer-specific survival [56]. Therefore, the authors conclude that anatomical radical retropubic prostatectomy continues to represent the gold standard in the surgical management of clinically localized Pca to which alternate treatment options should be compared.

Minimally invasive laparoscopic surgery and especially “robot-assisted laparoscopic radical prostatectomy” has also contributed a lot to the management of localized Pca. Robot-assisted radical prostatectomy (RARP) is gaining increasing acceptance among urologists and especially among patients because of widespread advertisements, and it has become the dominant technique in the United States despite a paucity of prospective studies or randomized trials supporting its superiority over RRP [57]. Although there is no prospectively randomization with open radical prostatectomy, experts indicate that there is sufficient evidence in order to suggest RARP as a valuable therapeutic option for clinically localized PCa [57]. 

Further developments in robot assisted surgery such as single port surgery may add some advantages to the surgical management of localized Pca, but these techniques have some serious limitations such as a very long learning curve and lack of ideal instruments. With all these high technological advances, expectations of patients with localized Pca have also increased accordingly. These high expectations were recently summarized and analyzed as “Trifecta” and more recently as “Pentafecta” [58, 59]. Authors briefly believe that “Pentafecta (cancer control, continence, and potency, no postoperative complications, negative surgical margins)” outcomes accurately represent patients' expectations after minimally invasive surgery for Pca and that this definition is highly beneficial and can be used when counseling patients with clinically localized disease [59]. And more recently, an outstanding group of authors with high expertise in this field have introduced the survival, continence, and potency (SCP) classification in order to report the oncologic and functional outcomes [60]. 

## 10. Radiation Therapy

Another radical treatment option for Pca is radiation therapy [3]. Advances in technology are making it possible to  target  radiation for Pca more precisely than in the past [61]. Currently used methods such as conformal radiation therapy (CRT), intensity modulated radiation therapy (IMRT), and proton beam radiation allow to treat only the prostate gland and avoid radiation to normal surrounding tissues as much as possible. These methods are expected to increase the effectiveness of radiation therapy while reducing the side effects. Studies are being done to find out which radiation techniques are best suited for specific groups of patients with Pca. There are also many studies under process in order to improve the effectiveness of radiation therapy. So far, though, no study has  arised.  Recently, a linear accelerator (CT-linac) has been introduced to improve results of radiotherapy especially when prostate movements are problematic for intensity-modulated radiotherapy [62].

## 11. New Horizons: Focal Therapy

Another area of research is “focal therapy” for localized Pca. This approach attempts to mirror the evolution of breast cancer treatment, which often involves “lumpectomy” as part of the initial management of the disease. Similarly, “partial nephrectomy” for small renal masses also represent a logical model for focal therapy in localized Pca. Focal therapy involves treatment of only that part of the prostate that is affected by cancer and uses methods like cryotherapy, high intensity focused ultrasonography (HIFU), and brachytherapy (seed implantation) to treat the cancer [63]. Several energy modalities are being developed to achieve the trifecta of continence, potency, and oncologic efficiency [63]. Focal therapy is still at its infancy and its role is unclear because of unresolved problems related to the lack of a proper method for complete evaluation of cancer location within the prostate and the potential coexistence of many different cancerous areas within the same prostate. These alternatives are still considered to be “experimental” in guidelines [3]. However, with the advances in imaging and especially in MRI, this approach will find a special place between surveillance and radical therapies in the management of localized Pca. In a recent review, it was underlined that guidance of thermal therapies for focal ablation of Pca will likely prove critically dependent on MRI functioning in four separate roles, summarized as device positioning, thermal monitoring of prostate ablation, and depiction of ablated prostate tissue [64]. A fourth critical role, identification of cancer within the gland for targeting of thermal therapy, is more problematic at present but will likely become practical with further technological advances [64]. 

As a conclusion, the management of localized Pca has dramatically changed in the last decades. However, further revolutionary changes in the diagnosis and management of Pca are awaited in the near future. It may be difficult to define a worldwide accepted screening policy because of different health systems in each country but new markers will soon be available in the market in order to increase the specificity and sensitivity in the diagnosis of Pca. Investigators have focused on identifying and isolating the gene, or genes, responsible for prostate cancer, and this will obviously help us to understand the basics of Pca. There are several promising medical treatment options, which are already used or under investigation for the management of metastatic Pca. But researchers postulate that these new alternatives may get involved in the management of localized Pca in the future. In addition to investigations in order to prevent Pca, it is also clear that life style and diet modifications will help us to decrease the prevalence of Pca. Advances in diagnostic techniques will probably help us to define the disease in the earlier stage in a less morbid way and will probably let us decide whether to do active surveillance or perform treatment especially with minimal invasive focal treatment options in the majority of cases.
